# Surgeon or Bot? The Risks of Using Artificial Intelligence in Surgical Journal Publications

**DOI:** 10.1097/AS9.0000000000000309

**Published:** 2023-06-28

**Authors:** Ellen C. Shaffrey, Sahand C. Eftekari, Lee G. Wilke, Samuel O. Poore

**Affiliations:** From the * Division of Plastic Surgery, University of Wisconsin School of Medicine and Public Health, Madison, WI; † Department of Surgery, University of Wisconsin School of Medicine and Public Health, Madison, WI.

## Abstract

Mini-Abstract

ChatGPT is an artificial intelligence (AI) technology that has begun to transform academics through its ability to create human-like text. This has raised ethical concerns about its assistance in writing scientific literature. Our aim is to highlight the benefits and risks that this technology may pose to the surgical field.

Chat Generative Pretrained Transformer (ChatGPT) is a new language-processing artificial intelligence (AI) platform developed by OpenAI and released in November 2022. Since its release, the popularity of this new technology has exploded, given its ability to respond to complex requests instantly with human-like text. Its many capabilities include composing poems, writing code, explaining complicated topics, and writing essays tailored to a specific viewpoint or experience level (Fig. 1). Unfortunately, this technology’s functionality has led to it being used to assist in the production of academic literature and, as a result, has raised concerns about the ethics of AI use. In this *Surgical Perspective*, we aim to discuss the risks this new technology poses to the scientific community and the benefits it may provide to surgeon scientists.

**FIGURE 1.**
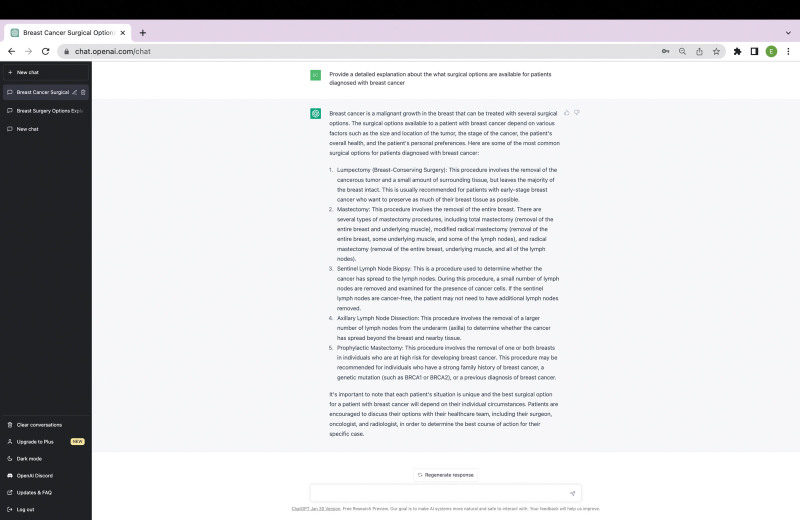
Example of ChatGPT’s ability to explain a complex surgical topic, in this case, the surgical options for patients diagnosed with breast cancer. ChatGPT indicates Chat Generative Pretrained Transformer.

Within the news, the earliest report of ChatGPT being implemented to assist with academic work was at the undergraduate level. By January 2023, colleges nationwide stated that students had been using ChatGPT to complete assignments, take finals, and write papers. A *New York Times* article highlighted that professors are redesigning courses to ensure the accurate assessment of student’s knowledge through oral tests or handwritten essays.^1^ Furthermore, honor codes are being updated, and the implementation of AI-generated text detection systems are being incorporated into universities’ armamentarium of tools to reduce plagiarism.^1^

In medicine, ChatGPT has already demonstrated an extensive knowledge base by passing all three United States Medical Licensing Exams (USMLE Step 1, Step 2CK, and Step 3) without specialized training or reinforcement.^2^ While the USMLE program stated that ChatGPT was not exposed to an utterly representative test, given that certain question types, such as those with pictures or clinical skill simulations were excluded, they noted that with the continued evolution of technology ChatGPT would likely be able to overcome these shortcomings in the future.^3^

This medical knowledge has been utilized to write publications with surprising authenticity. A study by Gao et al^4^ found that when blinded human reviewers were asked to differentiate between AI-generated and original abstracts, they incorrectly identified 32% of AI-generated abstracts as real and 14% of the original abstracts as AI-generated. Furthermore, all the AI-generated texts received a median originality score of 100% from a software-based plagiarism detector. This led to the conclusion that it was difficult to differentiate between the original and the AI-generated abstracts.^4^ However, ChatGPT’s data parameters are limited to information collected up to September 2021; therefore, they frequently provide incorrect or repetitive information, and their responses are more formulaic.^5^

Despite a report in *Nature* citing that ChatGPT had been listed as an author of at least 4 scientific articles in January 2023, there has since been an exponential increase in publications discussing its use.^6^ A current PubMed search with the terms, “ChatGPT” and “surgery” produced 68 results. Its continued growth is likely inevitable; however, at this time, many articles highlight that AI platforms are unable to fully comprehend the complex interplay of anatomy, physiology, and pathology within surgical sciences and multidisciplinary patient care.^7–9^ As this technology continues to develop, its implementation could lead to flawed investigation methods or false results, making evidence-based decisions difficult. For example, when ChatGPT was instructed to write an abstract on outcomes after nipple-sparing mastectomy in the context of radiation therapy, it did so in seconds (see Supplemental Video, http://links.lww.com/AOSO/A228). However, when reviewing the citations provided by ChatGPT, they do not actually exist.

Increased awareness by editors and reviewers is needed when reviewing submissions, along with clear rules regarding AI disclosure and the potential implementation of AI recognition software within journal crosscheck systems. *Nature* has established ground rules for AI use, stating that AI will not be accepted as an author in any research papers, and researchers must document any use of AI tools within their methods and acknowledgments.^10^ Alternatively, *Science* has stated that no text generated by ChatGPT or any other AI can be used in a scientific article.^11^ Going forward, each scientific journal needs to establish its own set of guidelines for the utilization of AI by clinician-scientists and researchers.

Nevertheless, ChatGPT is the first AI program that can create efficient processes for rapid data acquisition and analysis and has significant potential in medical care and scientific inquiry. It can save time in completing ancillary tasks such as reading communications or emails, condensing information, or promoting ideas through content development. Furthermore, it could be used to assist with information gathering and manuscript editing. However, similar to the disclosures associated with company ownership or payments, the editors of surgical journals may need to create an AI disclosure to ensure that reviewers and readers know when and how these technologies were incorporated into a scientific submission.

While ChatGPT can theoretically improve writing efficiency, it does not substitute for human expertise, assessment, or hypothesis-driven scientific inquiry. We hope that proactively raising awareness of this evolving technology to the *Annals* readership will not only help develop strategies to identify and mitigate the use of ChatGPT in scientific writing but also lead to a discussion on how to utilize it to further the field of surgery and surgical science.

## ACKNOWLEDGMENTS

E.C.S.: Surgical perspective question development ideation, preparation of figures, writing the manuscript, critical review and approval of the manuscript, accountability for the contents of the manuscript. S.C.E.: Surgical perspective question development ideation, editing the manuscript, critical review and approval of the manuscript, accountability for the contents of the manuscript. L.G.W.: Surgical perspective question development ideation, writing the manuscript, critical review and approval of the manuscript, accountability for the contents of the manuscript. S.O.P.: Surgical perspective question development ideation, editing the manuscript, critical review and approval of the manuscript, accountability for the contents of the manuscript.

## Supplementary Material


